# Borderline personality disorder in young people: associations with support and negative interactions in relationships with mothers and a best friend

**DOI:** 10.1186/s40479-021-00173-7

**Published:** 2022-01-06

**Authors:** Christel J. Hessels, Tessa van den Berg, Sofie A. Lucassen, Odilia M. Laceulle, Marcel A. G. van Aken

**Affiliations:** 1 Centre of Expertise on Early Intervention HYPE, GGz Centraal, PO Box 3051, 3800 DB Amersfoort, The Netherlands; 2 Department of Developmental Psychology, Utrecht University, Utrecht, The Netherlands

**Keywords:** Borderline personality disorder, Young people, Relationships, Mothers, Best friend

## Abstract

**Background:**

Impaired interpersonal functioning has been highlighted as a core feature of borderline personality disorder (BPD). Adolescence and young adulthood form important developmental stages within both the emergence of BPD and the development of interpersonal functioning, which takes place mostly in relationships with parents and friends. This study aimed to: (i) investigate relations between BPD symptoms and both supportive and negative interactions with mothers and best friends; (ii) investigate whether the relations were moderated by age; (iii) test the robustness of our findings by comparing the results based on self-reports with results from a subsample in which supportive and negative interactions with mothers were rated by the mother.

**Methods:**

312 young people referred to mental healthcare completed self-report measures on BPD and supportive and negative interactions. Multiple regression analyses were conducted to examine the relations between BPD features and perceived supportive and negative interactions with mothers and a best friend, and to investigate whether these relations were moderated by age. Robustness of our findings was studied in a subsample (*n* = 104), by using a multi-informant design in maternal report on supportive and negative interactions with mothers.

**Results:**

Multiple regression analyses demonstrated that negative interactions with mothers as well as with a best friend were related to more BPD symptoms in young people. Supportive interactions were not related to BPD symptoms. Both BPD and quality of relations were not related to age. In a subsample in which supportive and negative interactions with mothers were rated by the mother, the maternal report showed slightly different results. In this model, both supportive and negative interactions with a best friend were positively related, whereas interactions with mothers were not related to BPD symptoms in young people.

**Conclusions:**

Results highlight the importance of relationships with mothers and a best friend during adolescence and young adulthood. Given that BPD often emerges during this developmental phase, future research is needed to clarify how quality of relationships could alter pathways toward BPD in young people.

**Trial registration:**

Not applicable.

## Background

In the last decennia, the reluctance and ambivalence about assessing borderline personality disorder (BPD) in young people has shifted to an increasing focus on adolescence and early adulthood as the developmental phases when BPD commonly has its onset [1]. BPD is defined by high comorbidity and poor outcomes [2]. BPD has been associated, early in the course of the disorder, with high levels of social impairment [3], such as poorer general psychosocial functioning, poorer peer relationships and problems with family relationships [4], and impairments in theory of mind and mentalizing [5]. Moreover, research has shown that BPD in young people has a unique predictive value for poor psychosocial functioning, above and beyond mental state disorders and other personality disorder diagnoses [3, 4]. Even at a subthreshold level, the criteria of BPD are associated with poorer social and occupational functioning in adolescence and young adulthood [6], as well as in adulthood [7, 8].

The diagnosis BPD is not a fixed diagnosis, and the symptoms wax and wane during development. In adolescents, individual changes in psychosocial functioning appear to be related to changes in BPD symptoms, as an increase of BPD symptoms was related to an increase in psychosocial dysfunction, while a decline of BPD symptoms was related to improvement of psychosocial functioning [9]. This is an important finding, given that adolescence is considered a key developmental period concerning the onset of BPD. During adolescence and young adulthood, full threshold and subthreshold BPD may interfere with the process of gradually assuming more adult roles and responsibilities, which are necessary for adequate interpersonal functioning. Problems in interpersonal functioning are considered a central problem in BPD as well as in personality pathology in general [10], and in contrast to the relatively unstable nature of the diagnosis BPD, both in adolescents and in adults, problems in social functioning are relatively stable and may have long-lasting consequences for the individual’s functioning [11]. Social and interpersonal functioning develop in the context of social relationships. Therefore, social relationships can be seen as the key element of understanding the course of BPD [12]. This suggests that a better concept of relationships in relation to BPD in adolescence and young adulthood is needed in order to grasp the context of development of possible impairments in social functioning better.

### Social relationships in young people with BPD

During adolescence and young adulthood the development of social autonomy, establishing intimate relationships, and finding a new balance in the relationship with parents are important developmental tasks [13]. The relationships of adolescents and young adults with both threshold and subthreshold BPD can be challenged. Relatives, partners and friends of adults with BPD report considerable elevated objective and subjective burden and mental health problems, including depression and anxiety [14]. Similarly, families and friends of young people with BPD features report elevated levels of distress, negative caregiving experiences, and family environments high in expressed emotion, such as criticism and emotional overinvolvement, also when compared to adults in the general population or families and friends of young people with other serious illnesses [15]. In addition, reciprocal relations between BPD symptoms and parenting factors in adolescent girls in the community have been found, indicating that parenting may affect subsequent BPD symptoms and vice versa [16].

Considering the current social relationships with parents, it is found that adolescents who are developing personality disorders are more likely to experience conflicts with family members throughout the transition to adulthood [17]. In turn, persistent conflict with family members may have an adverse impact on psychosocial development throughout this important transitional period. Different explanations have been proposed for the findings that personality disorder traits were associated with both elevated contact and elevated conflict with family members [17]. One of the hypotheses the authors stated was that due to social skills deficits and interpersonal conflict adolescents with personality disorders may find it difficult to maintain satisfying relationships with others outside the family circle. In addition, they may tend to maintain frequent contact with family members during the transition to adulthood because they need sustained support from the family. This raises the question whether BPD, in addition to challenges in relationships with parents, is also associated with challenges in the relationships with friends during adolescence and young adulthood.

As the importance of parental physical presence decreases, relationships with peers are increasingly taking over functions of the relationship with parents, including intimacy, advice on behaviours and feelings, and social influence [18, 19]. Friendships are particularly important for socialization towards more mature roles in late adolescence and early adulthood, and play an important part in adaptive social development. Indeed, in a review Brechwald and Prinstein [20] have shown that in comparison to risky peer influence, healthy peer socialization processes can provide potential protection from maladaptive outcomes. Especially in adolescence, beyond friendships, the closeness elicited in *best friends* provides the context in which intimacy, trust, and emotional support are established and tested [21].

BPD features are consistently associated with problematic functioning across facets of peer functioning, such as friendship quality, peer victimization and bullying, and peer aggression [22]. Friendships during adolescence can be characterized by intense intimacy, and conflict is likely to occur faster and have a bigger impact when contact in relationship is very frequent [23]. Close friendships appear to place special burdens during early adolescence, and the expectations for exclusivity of best friendship in particular may be a marker of BPD [24]. Although different findings in associations between parental and best friend relationship quality and BPD were reported [11, 22, 25], research is needed to consider the dynamics of exclusive “best friend” roles in relation to BPD [22].

Given the major (psycho) social changes key to adolescence and young adulthood in general, and the development of psychopathology (including BPD) in some young people specifically, it is important to increase our understanding of BPD in the context of social functioning within adolescence and young adulthood. Many types of psychopathology, including personality pathology and BPD specifically, have their onset in (early) adolescence. Based on the Children in the Community Study (CIC) [26], a developmental trajectory for personality pathology was suggested describing an onset in early adolescence. In addition, a peak in prevalence rates of BPD was observed around middle to late adolescence followed by a decline into adulthood [27, 28]. At the same time, normative changes in relational functioning occur in adolescence and young adulthood. Bowlby’s theory of attachment [29] states that in infancy and childhood, children are primarily attached to their parents because they can offer them security and protection. As children enter adolescence, the importance of physical protection and security decreases, and adolescents become more independent and start to explore their environment on their own [30]. During the transition to young adulthood (18–25 years) the scope of change and more independent exploration of life’s possibilities increases further, gradually leading to more enduring choices in love, work, and worldviews [31]. Throughout adolescence and young adulthood, internal representations of relationships which shape mental concepts of interpersonal behaviour in close relationships across the life span, are considered to become resistant to change and generalized to other close relationships [29, 32].

The literature paints a developmental picture on how friendship relationships may be seen as an extension of the attachment relationship with the parents developed during childhood. However, the developmental path from childhood until adulthood is not a straightforward pathway, and there are several gaps in the current literature. Firstly, research on BPD features and aspects of the relationships with mothers focuses mostly on maladaptive parenting, such as maternal abuse or neglect [20, 21], or affective behaviours during a discussion task [19, 22]. While findings from these studies provide general support for the importance of the parent-child relationship, it remains unclear which specific features of this mother-child relationship are related to BPD in young people. Moreover, although previous research has suggested that the experience of the parent-child relationship can differ substantially between parent and child [33], to the best of our knowledge associations between quality of relationships and BPD have not been studied yet from the perspectives of both the young person and the mother. Secondly, while the increasing importance of relationships with peers have been reported across the literature [22], few studies have integrated the findings on quality of parental and best friend relationships, leaving a gap in how the quality of both parental and best friend relationships might be associated with BPD. Third, although studies have focused on parental and best friend relationships in young people, it is unclear whether these associations are related to age during the developmental phases of adolescence and young adulthood.

### The current study

Taken together, gaining insight into the associations between BPD features and social relationships with mothers and a best friend in adolescents and young adults would provide a window into the context for the development of psychosocial functioning during a crucial phase in the onset of BPD. The current study aims to add to the literature by examining relations between the quality of relationships with both mothers and a best friend and BPD in young people, investigating the role of age in these relationships, and comparing the results on the quality of relationships with the mother as reported both by the young person and the mother.

We expect that less supportive interactions and more negative interactions with mothers are related to more BPD symptoms. Similarly, we expect that less supportive interactions and more negative interactions with a best friend are related to more BPD symptoms [20, 34]. With regards to the role of age, it is hypothesized that the link between maternal supportive and negative interactions and BPD becomes weaker when adolescents grow older. Thus, associations between the maternal factors (maternal supportive and maternal negative interactions) and BPD might be smaller for older individuals and larger for younger individuals (i.e., moderation). For relationships with best friends, the opposite pattern is hypothesized; that is, we expect that associations between the best friend factors (best friend supportive and negative interactions) and BPD might be larger for older individuals and smaller for younger individuals.

Finally, in addition to studying the quality of parental relationships from a young person’s perspective, a parent’s perspective on this relationship can add to a more informative view by understanding individual differences between youths’ and parents’ reports of family relationships. Therefore, we will test the robustness of our findings by comparing the results based on self-report with results from a subsample in which supportive and negative interactions with mothers were rated by the mother.

## Methods

### Participants and procedures

This study is part of an ongoing clinical cohort study on BPD features in young people referred to mental health care (BPD Young). The sample consisted of 341 adolescents and young adults between 12 and 26 years old who were referred to specialized mental health care services for assessment and treatment of psychiatric problems, such as anxiety disorders, mood disorders, and personality pathology. Of the 341 participants in the study, 312 had data on all study variables and were included in the analyses. The final sample consisted of 214 females (69%) and 97 males. One participant did not identify themselves as either boy or girl. The ethnicity of the sample was identified by the native language of the participants. Of the 312 participants, 294 participants (94%) reported Dutch as their native language. Other reported ethnicities were English (*n* = 6), Turkish (*n* = 3), Spanish (*n* = 3), Berber (*n* = 1), or other, not specified (*n* = 5). When looking at daytime activities, 238 participants (76%) were currently enrolled in education, such as post-secondary vocational education (24%), higher general secondary education (15%) or higher vocational education (14%). Of the participants who were not enrolled in education, 41% had a job (*n* = 30). In addition, data were collected from mothers of the participants (*N* = 114), including biological mothers, foster mothers, and stepmothers. Of the 114 mothers in the study, 104 had data on all study variables and were included in the analyses. Data were collected between August 2018 and December 2020. Mean scores and standard deviations of age, construct BPD of the SCID-II-PQ and the NRI-BSV subscales are shown in Table 1. To provide a full picture on the dimension of scores on the SCID-II-PQ-BPD and in a representative sample of young people referred to mental health care, we did not exclude participants who had 0 BPD features (3 participants; 0.96%).

**Table 1:** Descriptive Statistics for the Main Study Variables

	Range	Mean	SD
Age	12.00–26.00	17.79	3.03
BPD (SCID-II-PQ-BPD)	0.00–15.00	8.92	3.77
*NRI -BSV Young person’s Perspective*			
Supportive Interactions Mother	1.00–5.00	2.76	0.89
Neg Interactions Mother	1.00–5.00	2.32	0.91
Supportive Interactions Best Friend	1.00–5.00	3.32	0.90
Neg Interactions Best Friend	1.00–4.33	1.51	0.51
*NRI -BSV Mother’s Perspective*			
Supportive Interactions Mother	1.47–4.00	2.51	0.54
Neg Interactions Mother	1.00–4.56	1.93	0.68

The measures for the study were part of the structured clinical assessment at entry to mental health care organizations in the Netherlands. Informed consent was obtained from patients (and caregivers when the patient was below 16 years of age), and patients agreed that the data could be used anonymously for research purposes. The study was approved by the Faculty Ethics Committee of the Faculty for Social and Behavioural Science of Utrecht University and the Institutional Research Board (FETC17–090). A research assistant was available, either physically or online, during the questionnaire assessment for the young person. Mothers completed the questionnaires online.

### Measures

#### BPD features

BPD features were operationalized with the Borderline scale of the SCID-II screening questionnaire (SCID-II PQ-BPD [35, 36]). The SCID-II PQ-BPD is a screening self-report questionnaire that consists of fifteen items in a yes/no response format. Items correspond to the nine DSM-IV BPD criteria. Each DSM-IV criterion has one question, except for criterion three (identity disturbance; four questions), five (recurrent suicidal behaviour; two questions), and eight (inappropriate anger; three questions). A BPD-score was calculated by counting the number of the affirmative answered items. Different studies showed that the SCID-II PQ-BPD was reliable in outpatient youth (*α* = .88 [36]; *α* = .85 [37]), but found different cut-off scores to obtain the best value of sensitivity and specificity predicting 5 or more criteria of BPD according to DSM-5. Chanen and colleagues [36] found a cut-off score of 12, while results in a comparable Dutch clinical sample of young people indicated a cut-off score of 6 with the best sensitivity and specificity [37]. Research indicates that in outpatient youth, the SCID-II PQ-BPD has satisfactory psychometric qualities [36]. That is, the instrument has a moderate sensitivity, high specificity, and moderate to high predictive value. Compared to other screening questionnaires for BPD, the aforementioned study showed that the SCID-II PQ-BPD had the highest overall diagnostic accuracy, test-retest reliability, and internal consistency. The inter-item reliability of the scale was very good (α = .82).

#### Supportive and negative interactions with mother and a best friend

Both the young person’s and mother’s perception of social relationships were measured with a Dutch translation of the *Network of Relationship Inventory – Behavioural Systems Version* (NRI-BSV [38, 39]). The NRI-BSV assesses the extent to which young persons’ dyadic relationships with best friends and parents are each characterized by behaviours commonly involved in the attachment, caregiving, and affiliative behavioural systems. The questionnaire consists of 24 items, using a 5-point Likert-scale, ranging from 1= ‘*very little or not at all*’ to 5 = ‘*could not be more*’. Furman and Buhrmester [39] provided consistent support for two factors: Supportive and Negative Interactions, which were minimally related (*r* = −.22, *p* < .001 to *r* = .02, *p* = .86). The psychometric properties of the NRI-BSV have been found to be good: the internal consistencies of the factors were good. Cronbach’s alpha for these two factors were comparable to the findings of Furman and Buhrmester [39]. Internal consistency was good for supportive interactions with best friends (α = .95), negative interactions with best friends (α = .90), supportive interactions with mothers (α = .94), and negative interactions with mothers (α = .96). Furthermore, internal consistency was good for the maternal report on both supportive interactions (α = .83) and negative interactions (α = .94).

### Statistical analyses

Descriptive statistics and bivariate correlations were estimated for age, the four variables of the NRI-BSV, and BPD. Hierarchical regression models were used, in which BPD was regressed in separate blocks on: 1) age, gender, 2) the four variables of the NRI-BSV (maternal supportive interactions, maternal negative interactions, best friends supportive, best friends negative interactions), and 3) interaction terms of age × the four variables of the NRI-BSV. The interaction terms were separately added to the model. Finally, all steps were repeated with the variables maternal supportive interactions and maternal negative interactions from the mothers’ perspectives, instead of from the young persons’ perspectives. All analyses were carried out in R version 3.6.0.

## Results

### Bivariate associations

Correlations between social relationships and BPD are displayed in Table 2, with self-report below the diagonal, and supportive and negative interactions with the mother based on maternal report above the diagonal. Age was not related to any of the self-reported variables. However, age was negatively related to both supportive interactions with the mother and negative interactions with the mother, reported by mothers, suggesting less interactions between older participants and their mothers than between the younger participants and their mothers. Females had more supportive interactions with their mothers than males, reported by mothers, but not according to the self-reports. Females also reported more BPD features than males. Furthermore, more BPD features were related to both more supportive interactions and more negative interactions with the best friend. More BPD features were also related to more negative interactions with the mother, but not with supportive interactions, as reported by both youths and their mothers. Supportive interactions with a best friend were positively related to negative interactions with a best friend. Contrarily, supportive interactions with the mother were negatively related to less negative interactions with the mother, as reported by youths. However, no relationship between these variables was found from the maternal report.

**Table 2:** Pearson’s Correlations between Borderline Personality Disorder and Predictor Variables

	1	2	3	4	5	6	7
1 Age	–	.09	.07	−.22*	−.32**	−.06	.10
2 Gender^a^	−.02	–	.28**	.25**	.02	.24*	.11
3 BPD	.09	.24**	–	.05	.19*	.27**	.36**
4 Supportive Interactions Mother	.00	.11	−.06	–	.02	.20*	.01
5 Neg Interactions Mother	−.01	.10	.34**	−.22**	–	.01	.20*
6 Supportive Interactions Best Friend	−.08	.21**	.15**	.26**	.18**	–	.16
7 Neg Interactions Best Friend	.01	.03	.30**	.09	.23**	.16**	–

*Note.* Correlations between self-reported measures are shown below the diagonal (*N* = 312); a subsample is shown above the diagonal, in which supportive and negative interactions with the mother is reported by mothers (*N* = 104)

^a^For gender, 1 = male, 2 = female, 3 = other

*p < .05, **p < .01

### Multivariate associations

To investigate whether age and quality of relationships were related to BPD in young people, we regressed both age and gender (step 1), the variables supportive and negative interactions with both mothers and a best friend (step 2), and the interaction between age and the variables in step 2 on BPD (step 3). Results from self-report (Table 3) show that the model accounted for 21% of the variance in BPD scores. Results suggested that gender is a significant predictor of BPD, with females having more BPD features than males (β = .24), whereas age is not related to BPD. Of the quality of relationships variables, only negative interactions with both the mother (β = .25) and a best friend (β = .23) had a significant contribution to our model, implying that negative interactions, but not supportive interactions, are related to more BPD symptoms. The interaction variables did not have a significant contribution to the model, implying that these relations between the quality of relationships variables and BPD do not get stronger or weaker as patients are older.

**Table 3:** Regression Coefficients of the Investigated Relationships and Moderating Effects

Step 1	*B*	*SE B*	β	*P*	*R*^2^
Age	0.11	0.07	.09	.096	.06
Gender	1.91	0.44	.24	**.000**	
Step 2	*B*	*SE B*	β	*P*	*R*^2^
Age	0.12	0.06	.10	.058	.21
Gender	1.62	0.42	.20	**.000**	
Supportive Interactions Mother	−0.27	0.23	−.06	.252	
Neg Interactions Mother	1.03	0.23	.25	**.000**	
Supportive Interactions Best Friend	0.21	0.23	.05	.363	
Neg Interactions Best Friend	1.73	0.39	.23	**.000**	
Step 3	*B*	*SE B*	β	*P*	*R*^2^
Age	0.00	0.21	0.00	.990	
Gender	1.58	0.42	.20	.000*	
Supportive Interactions Mother	−1.05	1.28	−.25	.413	
Neg Interactions Mother	1.03	0.23	.25	.000*	
Supportive Interactions Best Friend	0.21	0.23	.05	.368	
Neg Interactions Best Friend	1.70	0.39	.23	.000*	
Age × Supportive Interactions Mother	0.04	0.07	.21	.536	.21
Age × Neg Interactions Mother	−0.06	0.07	−.27	.371	.21
Age × Supportive Interactions Best Friend	−0.04	0.07	−.20	.534	.21
Age × Neg Interactions Best Friend	0.05	0.11	.13	.671	.21

*Note*. In Step 3, all interaction effects were added to the model separately. Main effects in Step 3 were in this table only reported from the model with the interaction effect Age × Supportive Interactions Mother, because these main effects did not differ substantially across the models with interaction effects. Abbreviations: *B* = Unstandardized beta, *SE* = Standard error for the unstandardized beta, β = Beta, *P =* Propability, *R*^2^ = proportion of explained variance.

*p < .05.

Results from a subsample in which supportive and negative interactions with mothers were based on the maternal report (Table 4) show that the model accounted for 19% of the variance in BPD features. Results from the maternal report suggested that both supportive interactions and negative interactions with the mother are not significant predictors of BPD features. However, in this model, both supportive interactions (β = .19) and negative interactions (β = .26) with a best friend had a significant contribution to the model. This implies that youths with more BPD features have both more supportive and more negative interactions with best friends than youths with less BPD features. The interaction variables did not have a significant contribution to the model, implying that these relationships between the quality of relationship variables and BPD do not get stronger or weaker as patients grow older.

**Table 4:** Regression Coefficients of Investigated Relationships and Moderating Effects, Interactions with Mothers based on Maternal Report

Step 1	*B*	*SE B*	β	*P*	*R*^2^
Age	0.10	0.20	.05	.620	.06
Gender	2.39	0.83	.27	.005*	
Step 2	*B*	*SE B*	β	*P*	*R*^2^
Age	0.18	0.21	.08	.394	.19
Gender	1.75	0.83	.20	**.**037*	
Supportive Interactions Mother	−0.21	0.75	−.03	.782	
Neg Interactions Mother	1.02	0.60	.17	.091	
Supportive Interactions Best Friend	0.91	0.45	.19	.046*	
Neg Interactions Best Friend	1.86	0.66	.26	.006*	
Step 3	*B*	*SE B*	β	*P*	*R*^2^
Age	0.29	0.80	.14	.715	
Gender	1.77	0.84	.20	.038*	
Supportive Interactions Mother	0.53	5.16	.07	.918	
Neg Interactions Mother	1.01	0.61	.16	.101	
Supportive Interactions Best Friend	0.90	0.45	.19	.050	
Neg Interactions Best Friend	1.87	0.67	.26	.006*	
Age × Supportive Interactions Mother	−0.05	0.33	−.10	.885	.18
Age × Neg Interactions Mother	−0.22	0.25	−.52	.375	.19
Age × Supportive Interactions Best Friend	−0.06	0.19	−.21	.763	.18
Age × Neg Interactions Best Friend	0.28	0.27	.68	.309	.19

*Note.* Supportive and negative interactions with mothers were reported by mothers. All other variables were reported by youths themselves. In Step 3, all interaction effects were added to the model separately. Main effects in Step 3 were in this table only reported from the model with the interaction effect Age × Supportive Interactions Mother, because these main effects did not differ substantially across the models with interaction effects. Abbreviations: *B* = Unstandardized beta, *SE* = Standard error for the unstandardized beta, β = Beta, *P =* Propability, *R*^2^ = proportion of explained variance

*p < .05

## Discussion

The purpose of this study was to (i) evaluate the degree to which supportive and negative interactions with both the mother and a best friend were related to borderline personality disorder (BPD) features in youths, (ii) examine whether the relation between both supportive and negative interactions in mothers and best friends and BPD features was moderated by age, and (iii) study the robustness of our findings by comparing the results based on self-reports with results from a subsample in which supportive and negative interactions with mothers were rated by the mother.

Considering our first research aim, this study demonstrated that negative interactions with both their mother and their best friend were positively related to BPD features in youths. Youths with more BPD features reported more negative interactions with their mother and a best friend, but no differences were found in relation to supportive interactions with their mother and a best friend. These findings are interesting as social support is an important aspect of quality of relationships and is consistently linked to good mental health [40]. In addition, parental support is a protective factor for different manifestations of psychopathology [41], and specifically in mother-daughter relations positive maternal affective behaviours and positive dyadic affective behaviours seem associated with decreases in females’ BPD severity over time [34]. Specifically, considering non-suicidal self-injury (NSSI), which is considered to be a key precursor for BPD [11], supportive interactions such as family support and social connectedness can facilitate the cessation of NSSI [42]. These findings could imply that although supportive interactions are linked to good mental health, in help-seeking youths negative interactions have a more impetuous impact on BPD in youths, while the role of supportive interactions is more complicated.

The relations between BPD and negative interactions were consistent with evidence suggesting that problems in interpersonal functioning are a central problem in BPD [10], and more specifically confirm literature on relations between BPD features and perceptions of relationship quality in adults. Due to the cross-sectional nature of our study, this raises questions regarding which impact negative interactions have with the developmental pathway of BPD. Negative interactions might be a risk factor, a feature, or a consequence of BPD. Long-term follow up studies investigating the role of quality of relationships from childhood on towards adolescence and adulthood in relation to BPD would be important to further investigate their role in within the developmental pathway.

The relation between BPD features and relationship quality is suggested to be most important in those specific relationships in which people interact frequently [43, 44]. The findings add to the literature by investigating the same constructs in interpersonal functioning in different relationships highly relevant for young people, i.e. supportive and negative interactions with the mother and a best friend. In addition, the sample of an at-risk adolescent and young adult population allows findings to be generalized to young people referred to mental health care. For this reason we did not exclude participants who had 0 BPD features (3 participants; 0.96%).

Considering our second research aim, the results showed that age was not related to quality of relationships with the mother or a best friend, or to BPD features. In addition, associations between quality of relationships and BPD features did not differ for different ages. This is a noteworthy finding, as longitudinal studies with community samples suggest a peak in BPD features in early adolescence and then show a pattern of decline over time [26, 45], which was not found when looking at age differences in this clinical sample. This might imply that our sample is more similar to the subgroup of young people whose personality pathology tends to persist as they enter adulthood [28]. Alternatively, it may be that SCID-II-PQ-BPD is insufficiently sensitive when it comes to capturing age variance in adolescence and young adulthood [46, 47]. This should be studied more thoroughly to grasp a full developmental perspective of adolescence and young adulthood as important phases in the development of BPD. Considering the development of relationships, the literature paints a picture that during the phase of adolescence and young adulthood relationships normatively develop relying on parent support in early adolescence, followed by the start of developing their own identity, and entering into new interpersonal relationships such as best friends in later stages of adolescence [13, 30]. That we did not find age differences in quality of relationships with mothers and best friends could imply that the normative developmental pathway during adolescence and young adulthood might have a different course in a high risk sample for both the development of BPD features and quality of relationships. It could be that in addition to the impact of negative interactions, the developmental tasks in relational functioning get delayed till after young adulthood, which could be in line with the negative outcomes in the transition to adulthood associated with BPD in young people [48]. More research is needed to further study psychosocial and relational functioning in young people at-risk for BPD, also to distinguish potential factors which could influence this development further in treatment of young people.

Considering our third research aim, by adding the results of the mothers’ reports of quality of relationships with the mother, a slightly different picture appeared. Results demonstrated that in this model, both supportive and negative interactions with the best friend were positively related to BPD features in youth. However, no differences were found in relation to both supportive and negative interactions with their mother. These findings may be interesting for several reasons. Firstly, the findings based on the mother’s report, seem in contrast with both the self-report by the youth and evidence suggesting that young people with BPD experience elevated conflict and contact with their parents [17]. These findings might be seen as that youth might interpret the quality of social relations more negatively as part of their BPD, as many of the BPD features, such as fear of abandonment, unstable intense relationships, and affective lability, are likely to relate to how interpersonal relationships are perceived. Secondly, the finding that more BPD features in youth were related to both more supportive interactions and more negative interactions with their best friend seems noteworthy. Since we found that supportive interactions with a best friend were positively related to negative interactions with a best friend, this suggests that in young people at risk for BPD the experienced interactions with a best friend are more extreme and a best friend is supremely important. This could imply that a best friendship provides both ground for supportive as well as negative interactions. These results could be interpreted as a confirmation of the special burdens related to close friendships that are seen during early adolescence. In the relationship with a best friend, the expectations for exclusivity of best friendship in particular may be a marker of BPD [24]. The results in this study not only apply to young adolescents, but also to older adolescents and young adults.

The results of this study show a different view from the youth’s perspective and the mother’s perspective on quality of relationships. This is an interesting finding, as studies on multiple informants’ reports of psychological phenomena commonly yield discrepant estimates. The divergence between reports of various aspects of family relationships—including conflict and the quality of the relationship—may signal key dynamics that affect young person–parent relationships and, therefore, the developmental outcome for the young person [49]. A pattern of diverging reports–young people reporting low positive relationships relative to parents– appears to be a potent marker for maladjustment in young people. For example, when young peoples’ reports on family relationships are more negative than parents’ reports—this predicts more internalizing problems in young people [50, 51]. In addition, when young people and parents agree on reports of high levels of parental acceptance, young people tend to display relatively low levels of symptoms of depression relative to other reporting patterns [52]. Given these findings, in future research it would be important to study the patterns on diverging reports by young people at risk for BPD and their mothers as a possible risk factor in the developmental course of BPD in young people. In addition, given the present findings and the importance of best friend relationships in this developmental stage, it would be important to include the patterns on diverging self-report and best friend report as well in future studies.

There are two important limitations to this study. Firstly, the present study used a cross-sectional design. This means that we cannot draw conclusions on the development of young people. Future work should adopt a longitudinal design to fully map long-term development of BPD from early adolescence into adulthood. And even in the context of cross-sectional design, various approaches should be considered to address the issue of developmental stage. In the current study we focused on age differences, while for example clinical staging [27] may also be included to account for a better understanding of the developmental pathway of BPD. Specifically, clinical staging could add differentiation of early or milder clinical phenomena from those that accompany illness progression and chronicity, and offer guidance in the application of appropriate and proportionate interventions. Long term follow-ups of the young people would make it possible to investigate whether negative interactions should indeed be seen as (precursor) factors contributing to the development of BPD, or if the relations might be the other way around. Consistent with the reciprocal nature of parenting and BPD features [53], it is critical to recognize whether parental affective behaviours are likely to be both a contributing factor in the developmental pathway of BPD and a response to BPD features in young people. Secondly, our reliance on a relatively small sample size for the mother’s report. As the mother’s report seems additive to the report of the young person, additional studies involving larger samples are necessary to replicate the present findings.

Despite these limitations, there are important implications from this study. First, this study relies on a clinical sample of help seeking youth, which allows the findings to be both generalizable and applicable to a vulnerable group of individuals with (emerging) BPD. A second strong point is that this study makes use of multi-informant design, which means that multiple relevant perspectives on an individual’s social relationship quality were highlighted. Lastly, an important feature of the current study is the use of the NRI-BSV, considering that participants use the same set of items to describe their relationships with two different members of their social networks (mother, best friend). Similar supportive and negative interaction scale scores were derived for the different relationships, making it possible to compare the associations of the different relationships with BPD.

The findings from this study may also have noteworthy clinical implications. It confirms the need for specific attention for the quality of relationships with mothers and best friends in young people with BPD features throughout the full developmental span of early adolescence, later adolescence, and young adulthood. Interventions should encourage skills in solving and repairing negative interactions in young people with BPD features. In addition, the use of multi-informant designs in order to cover all relevant perspectives on an individual’s social relationship quality is also important in assessment of the context for functioning and further development of youth, which could be a target for intervention within the treatment of BPD in young people.

## Conclusion

In conclusion, BPD features and quality of relationships with mothers and best friends are related in help-seeking youth. The current findings highlight the importance of negative interactions in particular – in contrast to support with mothers and best friends in young people at risk for BPD. Age did not appear to play a role in the link between quality of relations and BPD features. Future research is needed to enhance a better understanding of the developmental factors and the way the develop in young people at risk for BPD.

## Data Availability

The datasets used and analyzed during the current study are available from the corresponding author on reasonable request.

## Abbreviations

BPD: Borderline Personality Disorder
DSM: Diagnostic and Statistical Manual of Mental Disorders
NRI-BSV: Network of Relationship Inventory – Behavioural Systems Version
NSSI: Non-Suicidal Self-Injury
SCID-II PQ: Structured Clinical Interview for the DSM-IV-TR Axis II Disorders -Personality Questionnaire
